# Pediatric cardiac arrest in the emergency department: Outcome is related to the time of admission

**DOI:** 10.12669/pjms.35.5.487

**Published:** 2019

**Authors:** Ali Yurtseven, Caner Turan, Funda Karbek Akarca, Eylem Ulas Saz

**Affiliations:** 1 Ali Yurtseven, MD. Department of Pediatrics, Division of Emergency Medicine, Ege University School of Medicine, Izmir, Turkey; 2 Caner Turan, MD. Department of Pediatrics, Division of Emergency Medicine, Ege University School of Medicine, Izmir, Turkey; 3 Funda Karbek Akarca, MD. Associate Professor, Department of Emergency Medicine, Ege University School of Medicine, Izmir, Turkey; 4 Prof. Eylem Ulas Saz, MD. Department of Pediatrics, Division of Emergency Medicine, Ege University School of Medicine, Izmir, Turkey

**Keywords:** Emergency medicine, Pediatrics, resuscitation, Outcomes, Time of admission

## Abstract

**Objectives:**

Nights and weekends represent a potentially high-risk time for pediatric cardiac arrest (CA) patients in emergency departments. Data regarding night or weekend arrest and its impact on outcomes is controversial. The purpose of this study was to determine the relationship between cardiopulmonary resuscitation during the various emergency department shifts and survival to discharge.

**Methods:**

We conducted a retrospective, observational study of patients who had visited our Emergency Department for CAs from January 2014 to December 2016. Medical records and patient characteristics of 67 children with CA were retrieved from patient admission files.

**Results:**

The mean age was 54.7±7.3 months and 59% were male. Rates of survival to discharge 35% (11/31) within working hours’ vs. out of working hours 3% (1/36). Among the CAs presenting to the emergency department, the survival rates were higher for working hours than for non-working hours (OR: 37.6 (2.62-539.7), p: 008). The rate of return of spontaneous circulation within working hours was higher than that of non-working hours (71% vs.19%) (p<0.001). Patients who received chest compression for more than 10 minutes had the lowest survival rate (2%) (p<0.001), whereas better outcome was associated with in-hospital CA, younger age (less than 12 months) and respiratory failure.

**Conclusion:**

Survival rates from pediatric CAs were significantly lower during non-working hours. Poor outcome was associated with prolonged cardiopulmonary resuscitation, out of hospital CA and older age.

## INTRODUCTION

Cardiopulmonary resuscitation (CPR) is one of the most common lifesaving interventions rarely performed in Pediatric Emergency Departments (PEDs).^1^ There are numerous disease processes and clinical situations such as respiratory failure, shock, severe trauma and septic shock that may cause cardiac arrest (CA).^2^ Early recognition and management of those may prevent developing CA. Since resuscitation carries significant importance for critically ill children, appropriate training of all healthcare providers to perform CPR should be organized in all PEDs.

Approximately two-thirds of pediatric in-hospital CAs (IHCAs) achieve return of spontaneous circulation (ROSC), and 20-40% of them survive to hospital discharge with favorable neurologic outcomes^.3^ Pediatric Out of hospital CAs (OHCAs) have the worst outcomes. Survival to hospital discharge typically occurs for <10% of these children and many develop severe neurologic sequelae.^4^ The pediatric OHCA population account for only 1% of all OHCAs.^5^

Critical illness and CA in the PEDs are rare. Cardiac arrest has an overall incidence of 8-20 cases per 100,000 children per year.^2^,^3^ The National Registry of Cardiopulmonary Resuscitation report showed that, out of 23,535 in-hospital CAs recorded over a 2-year period, only 778 (3,3%) occurred in children. Of those in children, only 8.9% occurred in the PED.^6^ There are limited data investigating management and outcomes of pediatric CA in the PEDs.

Although non-working hour shifts represent a potentially high-risk time for pediatric cardiac arrest in PEDs, data regarding night shift or weekend shifts CA and its impact on outcomes is controversial.^7^

This study was performed to determine the rate of survival to hospital discharge in children who received CPR and its relationship with the various PED shifts. It also aimed to demonstrate the first Turkish pediatric CA data conducted at PED.

## METHODS

**Fig.1:**
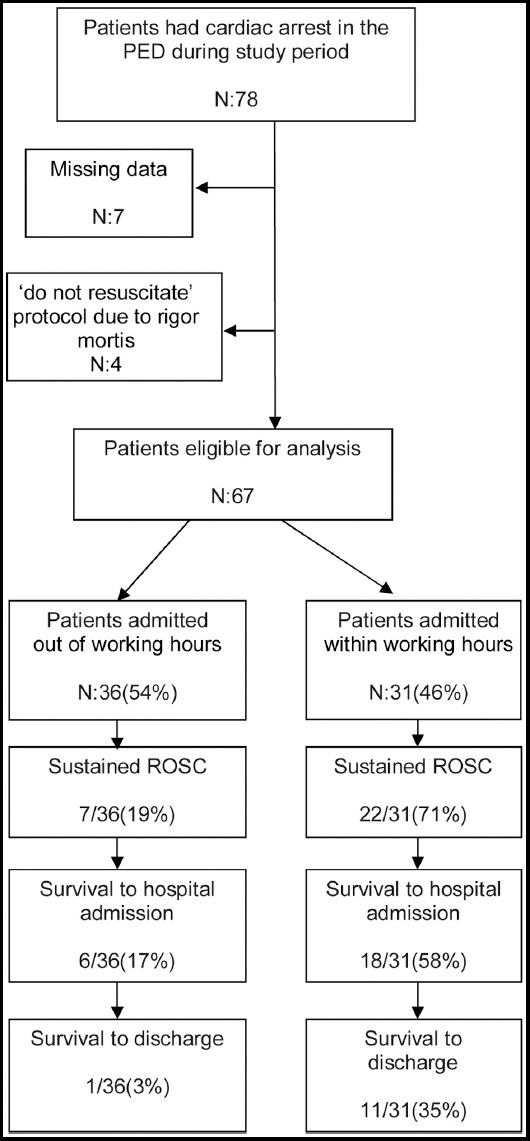
Diagram of utstein outcomes stratified by night or weekend shifts versus weekday shift.

This retrospective, single-center cohort study was conducted in a tertiary PED between January 2014 and December 2016. Seventy-eight patients who received CPR in our PED during the study period were included (Fig.1). We excluded four patients who had rigor mortis (“do not resuscitate” protocol) and seven patients due to missing data. Descriptive statistics were used to define the patients’ demographics, CA details and outcome. Study groups were classified into four groups based on age: infants (0–1year), toddlers (1–5 years), school children (5–10 years) and older children (10–18 years).

This study was approved by the local ethics committee. To maintain patient confidentiality, the forms did not include any data that would have enabled the identification of any patients. The procedures performed in this study followed the ethical standards in the Helsinki Declaration of 1964, as revised in 2008, as well as the national law.

In our country, pediatric emergency medicine (PEM) has been officially recognized as a subspecialty by 2011. Our PED is a tertiary-care teaching center and receives 70,000 visits annually. The PED is served by one PEM attending faculty, two PEM fellows, and six to ten rotating residents per month. Residents who work in our PED are supervised by PEM physicians (PEM attending staff, PEM fellows) during weekday shift, by general pediatricians during night shift or weekend shifts. Weekday hours in our PED are from 8 a.m. to 5 p.m., night shift is from 5 p.m. to 7:59 a.m. and weekend shift is from 17:00 p.m. on Friday to 7:59 a.m. on Monday.

Data were collected using a standard data collection form designed by the authors. This form included information on clinical and demographic characteristics of all study patients, age, sex, medical history, comorbidity, the main causes of CA, CPR duration, at the time of PED admission, location of CA, initial rhythm, transport by the emergency medical service (EMS) and the outcome of patient’s ROSC, discharged from the hospital and neurological status. All the remaining required data was retrieved from electronic medical records.

The primary outcome was determined as survival to hospital discharge. The secondary outcome was measured as sustained ROSC for, at least, one hour. Patients with unknown or missing survival status were excluded from analysis for both ROSC and survival to discharge.

All analyses were performed with SPSS for Windows (ver. 21.0 SPSS Inc., IL, USA). As potential factors that could influence the outcome, we selected the patients’ age group, clinical features, presence of comorbidity, location of CA, initial rhythm, transport by the EMS, admission time to the PED and CPR duration, and all were comparatively analyzed through Fisher’s exact test, Student’s t-test and Mann–Whitney U test. Statistical analysis followed two steps. The first examined univariate associations between the outcome and the potential confounders. In the second step variables found to be statistically significant were entered in a multiple logistic regression model using the backwards stepwise likelihood ratio procedure. A two-tailed probability value (p) of less than 0.05 was considered significant.

## RESULTS

During the study period 210,000 patients visited our PED, 78 (37 per 100,000) had CA. A total of 67 patients were eligible for final statistical analysis. Fifty-nine percent was male (n=39) and the mean age was 54.7±7.3 months. Toddlers and infants were the most crowded groups with rates of, respectively, 40% and 33%. In the study group, 25 (52%) patients had chronic illnesses; 10 (15%) neurological diseases, 9 (13%) respiratory diseases, 7 (10%) cardiovascular disease, 5 (8%) inborn errors of metabolism diseases, 2 (3%) malignancies and 2 (3%) malnutrition. The median time of performed CPR was 29 minutes in all patients and 10 minutes in the ROSC group.

Characteristics of the arrest events by PED shifts are shown in Table-I. There was 47 OHCA and 20 IHCA. Demographics were similar in the two groups. Majority of events (n=36, 53.7%) occurred at night or weekend shifts, of those 86% were OHCA (31 out of 36). The mean duration of performed CPR was longer in night shift or weekend shifts when compared with weekday shifts (38 versus 20 minutes, p <0.001). The two most precipitating conditions for CPR in the present cohort were respiratory failure and trauma. Respiratory insufficiency was more frequent in the night shift or weekend shifts group (42% versus 26%, p= 0.019). The proportion of comorbidity, EMS transport and shockable rhythms were similar in the two groups. Most IHCA events (75%) more likely occurred at weekday shifts, whereas the majority of OHCA (66%) were admitted at night or weekends shifts.

**Table I:** Characteristics of the arrest events by emergency department shifts.

Variables	Night or Weekend shifts (n=36)	Weekday shifts (n=31)	P-value
***Sex (M/F)***	21/15	18/13	1.000
Age (months)	55±7	54±8	0.850
<1 year of age– no.(%)	11(35)	11(31)	0.795
CPR^a^ duration- minutes	38±14	20±17	<0.001
>10 min CPR	32(89)	15(48)	<0.001
*Preexisting conditions – no.(%)*			
Respiratory failure	15(42)	8(26)	0.019
Trauma	7(19)	9(29)	
Neurological diseases	1(3)	5(16)	
Cardiac failure	5(14)	1(3)	
Sepsis/shock	2(6)	3(10)	
Other or unknown	6(16)	5(16)	
Comorbidity (+)– no.(%)	18(50)	17(55)	0.807
Transport by the EMS^b^ – no.(%)	15(42)	17(55)	0.332
*Locations of arrest– no.(%)*			
OHCA^c^	31(86)	16(52)	0.003
IHCA^d^	5(14)	15(48)	
Defibrillation (+) – no.(%)	4(11)	3(10)	1.000

a Cardiopulmonary resuscitation,

b Emergency medical servise,

c Out-of-hospital cardiac arrests,

d In-hospital cardiac arrests

ROSC rates by PED shifts are shown in Table-II. During the study period, 29 (43.2%) patients with CA achieve ROSC, (n=22) 71% of those were resuscitated during working hours (Fig.1) and 55% were IHCA. OHCA and events occurring during the night/weekend shifts were less likely to achieve ROSC (p <0.001). As a precipitating condition for CPR, neurological diseases and presence of comorbidity were associated with a higher rate of ROSC (p=0.008 and p=0.001). Age younger than 12 months also had higher ROSC. Critical factors that influence ROSC rates include the other demographics, EMS transport and the initial electrocardiographic rhythm, which were similar in groups.

**Table II:** The return of spontaneous circulation related variables.

Variables	ROSC^a^(+) (n=29, 43%)	ROSC(-) (n=38, 57%)	P value
Sex (M/F)	15/14	24/14	0.454
Age (months)	36±9	69±12	0.024
<1 year of age– no.(%)	14(64)	8(36)	0.034
Diagnosis – no.(%)			
Respiratory failure	11(38)	12(31)	0.008
Trauma	2(7)	14(37)	
Neurological diseases	6(21)	0(0)	
Cardiac failure	2(7)	4(11)	
Sepsis/shock	3(10)	2(5)	
Other or unknown	5(17)	6(16)	
Shift hours – no.(%)			
Out of working hours	7(19)	29(81)	<0.001
Within working hours	22(71)	9(29)	
Comorbidity (+) – no.(%)	22(63)	13(37)	0.001
Transport by the EMS^b^ – no.(%)	11(34)	21(66)	0.218
Locations of arrest – no.(%)			
OHCA^c^	13(28)	34(72)	<0.001
IHCA^d^	16(80)	4(20)	
Defibrillation (+) – no.(%)	5(71)	2(29)	0.225

a Return of spontaneous circulation,

b Emergency medical servise

c Out-of-hospital cardiac arrests,

d In-hospital cardiac arrests.

Survival to discharge in our group is shown in Table-III. Only 12 (18%) patients in this cohort survived to discharge after CA. Children with CA that occurred at night /weekend shifts had a significantly lower survival rate than patients whose arrest occurred at working hours (multivariable-odds ratio [OR], 37.6 [95%CI, 2.62–539.7], p=0.008). Younger age (less than 12 months) and respiratory failure were significantly better predictors for survival to discharge. A better outcome was also observed in children who had arrest at the PED area than whose arrest occurred at non-hospital settings (discharge rate 40% versus 8% p= 0.002). The mean duration of performed CPR and the mean number of epinephrine administration were significantly shorter in the surviving group as expected when compared with the non-surviving group (6 minute versus 35 minutes, p <0.001) (1.7 versus 7.9, p <0.001) (Because the CPR duration and number of epinephrine administration have an absolute impact on outcome, they were excluded from the multiple logistic regression analysis).

**Table III:** Factors predicting survival to discharge.

Variables	Discharged(n=12, 18%)	Died (n=55, 82%)	Univariate analysis		Multivariate analysis	

			Odds ratio (95% Cl) P-value		Odds ratio (95% Cl)	P-value
Sex (M/F)	7/5	32/23	0.9 (0.28-3.52)	0.992	-	-
Age (months) (mean)	30±6	60±13		0.132	-	-
<1 year of age– no. (%)	7(32)	15(68)	3.7 (1.02-13.52)	0.038	1.5 (0.19-11.95)	0.698
CPR^a^duration (mean, min.)	6±2	35±16		<0.001	-	-
>10 min CPR	1(2)	46(98)	56.2 (6.43-491.5)	<0.001	-	-
Diagnosis – no. (%)				0.037		0.729
Respiratory failure	7(59)	16(29)	2.2 (0.36-14.25)	0.053	0.4 (0.03-5.74)	0.438
Trauma	0(0)	16(29)	-		-	-
Cardiac failure	1(8)	5(9)	5.0 (0.34-72.76)	0.934	0.9 (0.20-57.84)	0.977
Sepsis/shock	0(0)	5(9)	-		-	-
Other or unknown	1(8)	10(18)	10.0 (0.73-135.32)	0.404	4.6 (0.22-96.52)	0.322
Neurological diseases	3(25)	3(6)	Reference		Reference	
Shift hours – no. (%)						
Out of working hours	1(3)	35(97)	19.2 (2.31- 160.3)	<0.001	37.6 (2.62-539.7)	0.008
Within working hours	11(35)	20(65)				
Comorbidity (+) – no. (%)	9(26)	26(74)	3.3 (0.81-13.70)	0.081	-	-
Transport by the EMS^b^ – no. (%)	3(9)	29(91)	3.3 (0.81-13.70)	0.081	-	-
Locations of arrest – no. (%)	4(8)	43(92)	7.1 (1.83-27.92)	0.002		
OHCAc	8(40)	12(60)			3.8 (0.47-31.20)	0.205
IHCAd	2(29)	5(71)	1.8 (0.21-13.21)	0.600		
Defibrillation (+) – no. (%)	1.7±0.2	7.9±0.5		<0.001	-	-
Number of adrenalin (mean)						

a Cardiopulmonary resuscitation,

b Emergency medical servise ^c^Out-of-hospital cardiac arrests, ^d^In-hospital cardiac arrests

The most common identified terminal rhythm was asystole or pulseless electrical activity (PEA) and its frequency was 89.5% (n=60). Shockable rhythms were determined in 7 patients (5 ventricular fibrillation (VF) and 2 pulseless ventricular tachycardia (pVT)) and defibrillation was administered. Two of them were discharged from the hospital.

## DISCUSSION

This is the first national data with a study investigating the frequency and outcomes of pediatric CA in the PED setting. The reported incidence of pediatric CA in children and young adults ranges from 0.5 to 20 per 100,000 persons per year, which is less than our rate.^1^-^3^ This difference can be explained by location and some features of our center. Our PED is one of the busiest non-profit university/teaching hospitals in western Turkey, which is located at the heart of the 3^rd^ biggest city, Izmir. Since all of the subspecialties of pediatrics are covered in our hospital, we do medical care for complex patient’s emergencies and most OHCA occurred in urban municipality transports to our PED by the EMS.

Previous studies have found that the frequency of CA in infants was higher than other age groups in the pediatric population.^4^,^8^ In contrast, in this study we found that pediatric CA most likely occurred in toddlers. Many patients in this age group had comorbidity and follow up by our subspecialty outpatient’s clinic. In our cohort nearly half of the patients had chronic pre-existing conditions, which are consistent with some previous reports.^9^ The location of CA is the main reason for this wide range; OHCA episodes more likely occurred in patients who had lower comorbidity rate.

Numerous studies from developing and developed countries demonstrate that CAs in children frequently result from respiratory failure.^10^,^11^ However, some previous reports showed that cardiac conditions were the most common cause of CAs.^2^,^8^ In our study respiratory failure was also the most common cause of CA, followed by trauma. We believe that the mean age of the patients, the location of arrest episode and hospital/country type appear to have a significant influence on the differences.

Our most notable result was the higher rate of survival to discharge and ROSC during weekday shifts when compared with nights or weekends shift. These findings are consistent with previous studies data.^7^,^12^ The reason for poor prognosis of CA during the night shift might be multifactorial, potentially including healthcare staff and hospital staff as well as the differences in the etiologies of CA. Although etiologies were similar in our groups, most OHCA brought to the PED during non-working hours and most hospital staff had a lack of resuscitation experience. Residents or other physicians who work in PED at the night/weekend shift often lack the experience and skill needed in resuscitation. In our PED, PEM physicians manage patient’s weekday from 8 am to 5 pm, and night/weekend shifts are covered by subspecialty fellows and residents. The lack of resuscitation skills of residents in PED settings on basic and pediatric advanced life support (PALS) has been identified as a contributing factor to poor outcomes in pediatric CA. Timing and quality of CPR are among the most important factors to improve CA outcomes. Since the number of PEM physicians in our country is only around 45, it’s hard to cover all night/weekend PED shift for a while. Recent investigations have demonstrated that PALS training among healthcare providers may be associated with an increase in the rate of survival to discharge.^13^ This further shows that unwitnessed arrest, which is more common during the night shift, is related to less ROSC ratio.^14^,^15^

There are currently no firm guidelines regarding the duration of such resuscitation.^16^ In a large prospective multicentric study, Matos et al. found that the rate of survival to discharge and the probability of a favorable neurological outcome fell linearly in the first 15 minutes of CPR and decreased by 1.2% for each additional minute of chest compressions. They also showed that trauma patients had the worst outcome.^17^ In our cohort longer CPR durations were associated with poor outcomes for both survivals to discharge and ROSC. Mortality was more likely to occur in trauma patients and in patients who received CPR for more than 10 minutes. This finding supports Matos’ study.

Survival to discharge rates, the likelihood of favorable neurologic outcomes and functional status following CA vary substantially between OHCA and IHCA. These differences are based on many factors, including the predominant etiologies of IHCA and OHCA, the affected children and related comorbidities, the proximity to trained providers, and PED professionals and hospital staff. As expected our results had similarity with previous studies, which demonstrated that IHCA survival rate and any ROSC were higher than OHCA.^18^,^19^ Post-arrest care is one of the most important points for best outcome in cases of CA. Studies have demonstrated that, when coordinated, high-quality and comprehensive post-resuscitation care is provided, the rate of better outcome can be dramatically increased.^20^ Good outcomes in our IHCA group may be explained with effective post-resuscitation care.

It has previously been suggested that having chronic diseases was not significantly associated with ROSC and survival to discharge.^11^,^17^ Differently, we found that CPR due to neurological diseases and the presence of comorbidity was associated with higher rate ROSC. These results may be explained by well-trained parents who had children with special healthcare needs and already aware of emergency scenarios. When they feel like their children need emergency medical care they just call the ambulance or brought their children directly to PEDs.

In literature it has generally been reported that being infant was associated with more likelihood to have a poor outcome.^21^,^22^ Possible reasons for this are that patients younger than one year tend to have sudden infant death syndrome, unwitnessed arrests and non-shockable rhythm at the time of CA. Inconsistent with the previous studies, outcomes after CAs were better for infants in our study. The difference may be explained by a higher proportion of OHCA and trauma in children older than one year.

Only 12 (18%) patients in our cohort survive to discharge after CA. In this group good outcome predictors were IHCA, weekday shift arrests and non-traumatic conditions. Most recent studies on pediatric CA (OH, IH) in PED settings showed similar results and survival to hospital discharge rate was 12%–23%.^6^,^11^,^18^

### Limitations of the study

This is a retrospective study of the medical records obtained from the PED records; hence there may be an over- or under-estimation of these findings. We were unable to evaluate neurologic status because longer follow-up data were not available. We could not obtain the data about time to epinephrine administration. Moreover, the complications were not documented and it was not possible to assimilate properly whether the physicians were following all steps of CPR accordingly. It was conducted in a single center; therefore, its results cannot be generalizable to other settings.

## CONCLUSION

Unwitnessed arrests (especially OHCA) are more prevalent during the night/weekend shift; resuscitation during this shift may be associated with poorer outcomes independently of witnessing status. Due to higher IHCA rate infants had better outcome. Our data also demonstrated that early initiation of CPR with an experienced team (which works weekday) in a PED setting increased the discharge survival rate following resuscitation. Therefore, we emphasize that all medical staff should receive pediatric PALS course and CPR training with periodic renewal.
